# A Phylogenetic Analysis of the Brassicales Clade Based on an Alignment-Free Sequence Comparison Method

**DOI:** 10.3389/fpls.2012.00192

**Published:** 2012-08-29

**Authors:** Klas Hatje, Martin Kollmar

**Affiliations:** ^1^Abteilung NMR-Basierte Strukturbiologie, Max-Planck-Institut für Biophysikalische ChemieGöttingen, Germany

**Keywords:** Chaos game representation, Brassicales, *Brassica rapa*, phylogenetic tree, bootstrap re-sampling, frequency Chaos game representation

## Abstract

Phylogenetic analyses reveal the evolutionary derivation of species. A phylogenetic tree can be inferred from multiple sequence alignments of proteins or genes. The alignment of whole genome sequences of higher eukaryotes is a computational intensive and ambitious task as is the computation of phylogenetic trees based on these alignments. To overcome these limitations, we here used an alignment-free method to compare genomes of the Brassicales clade. For each nucleotide sequence a Chaos Game Representation (CGR) can be computed, which represents each nucleotide of the sequence as a point in a square defined by the four nucleotides as vertices. Each CGR is therefore a unique fingerprint of the underlying sequence. If the CGRs are divided by grid lines each grid square denotes the occurrence of oligonucleotides of a specific length in the sequence (Frequency Chaos Game Representation, FCGR). Here, we used distance measures between FCGRs to infer phylogenetic trees of Brassicales species. Three types of data were analyzed because of their different characteristics: (A) Whole genome assemblies as far as available for species belonging to the Malvidae taxon. (B) EST data of species of the Brassicales clade. (C) Mitochondrial genomes of the Rosids branch, a supergroup of the Malvidae. The trees reconstructed based on the Euclidean distance method are in general agreement with single gene trees. The Fitch–Margoliash and Neighbor joining algorithms resulted in similar to identical trees. Here, for the first time we have applied the bootstrap re-sampling concept to trees based on FCGRs to determine the support of the branchings. FCGRs have the advantage that they are fast to calculate, and can be used as additional information to alignment based data and morphological characteristics to improve the phylogenetic classification of species in ambiguous cases.

## Introduction

Phylogenetic analyses reveal the evolutionary derivation of species. A phylogenetic tree can be inferred from multiple sequence alignments of proteins or genes, which assume the conservation and contiguity over the total sample length between homologous sequences (Blair and Murphy, 2011). The alignment of whole genome sequences of eukaryotes is a computational intensive and ambitious task as is the computation of phylogenetic trees based on these alignments (Dewey, 2012). In particular, genetic recombination and shuffling during species evolution complicate whole genome alignments limiting species genome versus single gene, multiple gene, or transcriptome comparisons. However, it would be beneficial for the significance of the species trees, if also whole genome assembly data were taken into account. In the past two decades several methods have been suggested for alignment-free sequence analyses that mainly group into word (oligomer) frequency methods and methods that do not resolve the fixed word-length distance measures and are thus absolutely independent from the assumption of conservation and contiguity (reviewed in Vinga and Almeida, 2003). The latter category includes the Chaos Theory (Jeffrey, 1990) and the theoretical concept of Kolmogorov complexity (Li et al., 2001). More recent methods include the alignment-free estimation of the number of substitutions per site (Domazet-Loso and Haubold, 2009) and feature frequency profiles (Sims et al., 2009).

The Chaos Game Representation (CGR) denotes an algorithm, which produces fractal pictures and can be adapted to reveal patterns in DNA (Li et al., 2001) and even protein sequences (Basu et al., 1997; Pleissner et al., 1997). These CGR pictures exhibit the fractal property that the overall pattern of the CGR picture is repeated in smaller parts of the picture. It has been shown that this self-similarity even holds for whole genome sequences and its sub-sequences, like single chromosomes, contigs, or genes (Deschavanne et al., 1999; Almeida et al., 2001; Joseph and Sasikumar, 2006). Commonly, the pictures of DNA sequences are generated as squares such that the lower (A + T) and the upper (C + G) halves indicate the base composition and the diagonals the purine/pyrimidine composition. CGRs are unique descriptions of each DNA sequence and, in the case of whole genome sequences, can therefore be regarded as genomic fingerprints. However, the CGRs are not directly comparable. If the CGR pictures are divided into smaller squares by grid lines, each grid square represents the frequencies of the respective oligonucleotides as found in the whole sequence (Deschavanne et al., 1999; Almeida et al., 2001). These frequencies can be represented in Frequency Chaos Game Representation (FCGR) pictures with a gray scale to express the number of points within each grid square and with pictures for each length *k* oligonucleotide (with *k* = 1, 2, 3…). FCGRs are numerical matrices and can be used to infer phylogenetic trees based on distance methods (Wang et al., 2005). So far this approach has only been applied to reconstruct the phylogeny of 20 birds using nuclear genome data (Edwards et al., 2002), to analyze the mitochondrial genomes of 26 sample eukaryotes (Wang et al., 2005), and to sub-typing of HIV-I (Pandit and Sinha, 2010). One of the advantages of using FCGRs for phylogenetic reconstructions is that sequence, which cannot be aligned, can be used.

Here, we performed phylogenetic analyses based on three different types of data. Firstly we used the whole genomic sequence assemblies of all so far sequenced species in the taxon Malvidae, including that of *Brassica rapa*. Because a reference tree including all these species was not available we assembled and annotated all actin capping (CP) protein sequences (Cooper and Sept, 2008) and the sequences of the actin-related proteins Arp2 and Arp3 (Goley and Welch, 2006). These proteins are present in all eukaryotes and as single copies in the *Arabidopsis thaliana* genome. Thus they were not expected to exist in duplicates in the other analyzed species avoiding the ortholog-paralog problem. To infer the phylogeny of the different *Brassica* species, for which whole genome assemblies have not yet been produced, we used EST and mitochondrial genome DNA. The quality of the phylogenetic analyses depends on the resolution of the FCGRs (length of *k*) and thus on the length of the nucleotide sequences. Thus we only included those species for which a considerable number of EST clones were available. To estimate the support for the branchings, here, we apply the concept of bootstrap re-sampling to the comparison of FCGRs for the first time.

## Materials and Methods

### Data acquisition

The genome files were retrieved from diArk^1^ (Hammesfahr et al., 2011), and the mitochondrial genomes and EST reads from the NCBI database, each in FASTA format (Table 1). For the generation of the CGRs the contigs and reads of each dataset were concatenated. The whole genome assemblies as available from the sequencing centers contain both the nuclear and mitochondrial genomes, and potentially still some contaminations from other species’ DNA. However, given the sizes of the whole genome datasets, the contributions of the mitochondrial genomes and contaminating DNA to the FCGRs are negligible. The FCGRs of the whole genome data can thus be regarded as identical to the FCGRs of the nuclear genomes.

**Table 1:** **List of the species used in the analysis**.

Species	Whole genome			EST		Mitochondrial genome		
	Contigs	Nucleotides	Accession numbers	Reads	Nucleotides	Contigs	Nucleotides	Accession numbers
*Arabidopsis lyrata*	695	206667935	GL348713–GL349407					
*Arabidopsis thaliana*	5	119145879	NC_003070–NC_003071, NC_003074–NC_003076	1529700	400512451	1	366924	NC_001284
*Brassica rapa*	51658	273071614	AENI01000001–AENI01051658	213605	122970377	1	219747	NC_016125
*Capsella rubella*	853	134834574						
*Carica papaya*	3207	331271729	DS981520–DS984726	77393	54789864			
*Citrus clementina*	1128	295550349						
*Citrus sinensis*	12574	319231331						
*Eucalyptus camaldulensis*	274001	654922307	DF097775–DF126446					
*Eucalyptus grandis*	4952	691297852						
*Eutrema halophilum*	639	243117811		38022	20080214			
*Eutrema parvulum*	7	114396853	CM001187–CM001193					
*Gossypium raimondii*	1448	763818933						
*Theobroma cacao*	1782	351351221	FR720657–FR725448					
*Vitis vinifera*	33	486265422	FN597015–FN597047	446643	284204927	1	773279	NC_012119
*Brassica napus*				643437	381399492	1	221853	NC_008285
*Brassica oleracea*				179150	125257248	1	360271	NC_016118
*Limnanthes alba*				15331	8582959			
*Raphanus raphanistrum*				164119	104536170			
*Raphanus sativus*				150680	97973638			
*Tropaeolum majus*				10507	6436290			
*Brassica carinata*						1	232241	NC_016120
*Brassica juncea*						1	219766	NC_016123
*Lotus japonicus*						1	380861	NC_016743
*Millettia pinnata*						1	425718	NC_016742
*Ricinus communis*						1	502773	NC_015141

*The number of contigs/reads and the number of nucleotides for the whole genome, EST, and mitochondrial genome data files are given. In addition, for whole genome and mitochondrial genome data the NCBI accession numbers are given if available*.

### Implementation of the algorithm

The algorithm to calculate CGRs and FCGRs was implemented in C/C++. CGR positions were generated as lists in plain text and plotted for graphical presentations in the Scalable Vector Graphics (SVG) format^2^. Based on the CGR position values, FCGRs were calculated for each *k* in 1, …, 8. Distance calculations were implemented in Ruby^3^.

### Generating chaos game representations

**Figure 1:**
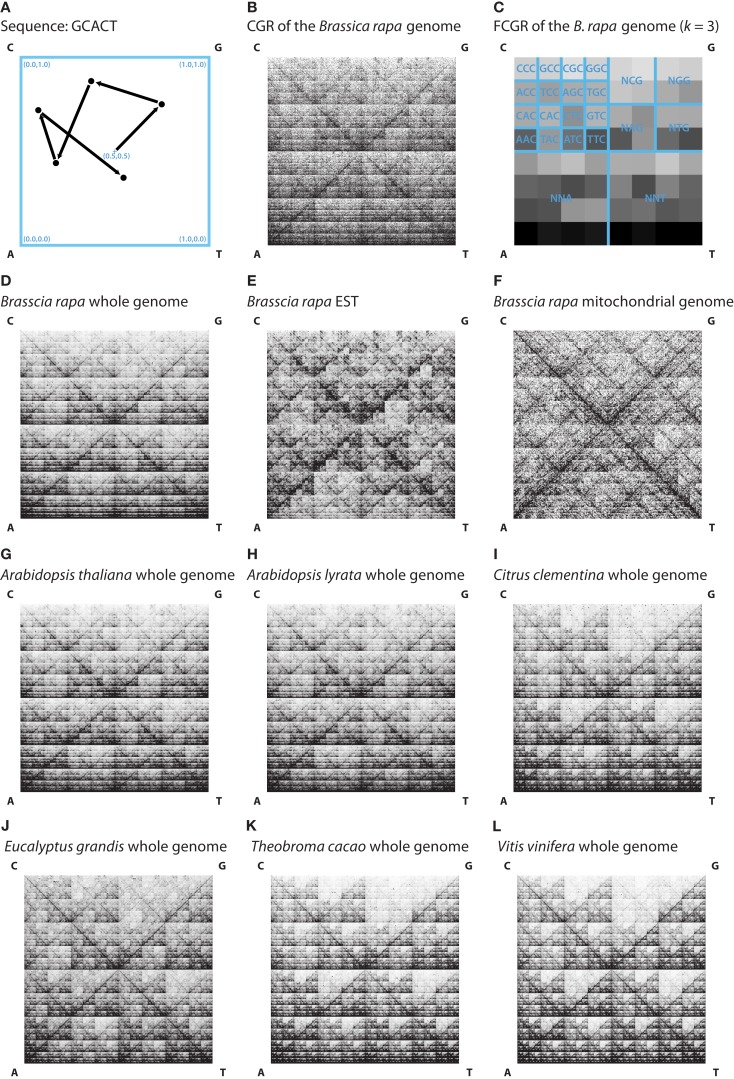
**(A)** A Chaos game representation (CGR) image is generated by drawing a unit square and, starting at the center (0.5, 0.5), plotting for each nucleotide of the sequence a point on half the distance to the corresponding vertex. In this example the CGR for the sequence “GCACT” was drawn. **(B)** The image shows the CGR of the first 1,000,000 nt of the *Brassica rapa* genome. **(C)** The figure shows an FCGR (*k* = 3) of the whole *Brassica rapa* genome illustrating the frequencies of points in the CGR in an 8 × 8 grid. The squares of the grid represent the occurrence of specific trinucleotides, which are labeled in the figure. In **(D–L)** the FCGRs (*k* = 8) of the whole genome **(D)**, EST **(E)** and mitochondrial genome sequences **(F)** of *Brassica rapa* and the FCGRs (*k* = 8) of the whole genome sequences of some representatives of the different clades **(G–L)** are shown for visual comparison.

Chaos game representations of the nucleotide sequences were generated by the following algorithm. A 1 × 1 square is drawn and each vertex labeled by a nucleotide. In agreement with other analyses we placed C in the upper left, G in the upper right, A in the lower left, and T in the lower right vertex. The starting point is defined as the geometric center of the square at position (0.5, 0.5). The respective nucleotide sequences are then plotted sequentially. For the first nucleotide a point is plotted on half the distance between the starting point (0.5, 0.5) and the vertex corresponding to this nucleotide. Subsequently for each following nucleotide a point is placed as mid-point between the previously plotted point and the vertex corresponding to the nucleotide (Figure 1A).

The algorithm can be expressed by the following equations:

(1) $$\begin{array}{c} \text{CG}{\text{R}}_{0}=\left(0.5,0.5\right) \end{array}$$

(2) $$\text{CG}{\text{R}}_{i}=\left\{\begin{array}{cc} \text{CG}{\text{R}}_{i-1}+0.5\cdot \left(\text{CG}{\text{R}}_{i-1}+\left(0.0,0.0\right)\right) & \;\text{if}\;\text{se}{\text{q}}_{i}=\;‘\text{C}\text{’}\\ \text{CG}{\text{R}}_{i-1}+0.5\cdot \left(\text{CG}{\text{R}}_{i-1}+\left(1.0,0.0\right)\right) & \;\text{if}\;\text{se}{\text{q}}_{i}=\;‘\text{G}\text{’}\\ \text{CG}{\text{R}}_{i-1}+0.5\cdot \left(\text{CG}{\text{R}}_{i-1}+\left(0.0,1.0\right)\right) & \;\text{if}\;\text{se}{\text{q}}_{i}=\;‘\text{A}\text{’}\\ \text{CG}{\text{R}}_{i-1}+0.5\cdot \left(\text{CG}{\text{R}}_{i-1}+\left(1.0,1.0\right)\right) & \;\text{if}\;\text{se}{\text{q}}_{i}=\;‘\text{T}\text{’} \end{array}\right.$$

The resulting plot is unique for each sequence. The overall pattern of points is repeated in each sub-square of the plot (Figure 1B). In addition, each plot based on a sub-sequence of the whole sequence has a similar appearance. Thus similar sequences result in similar CGR plots. Figure 1B shows the CGR of the first 1,000,000 nt of the *B. rapa* genome sequence.

The calculation of the frequencies of points within each sub-square results in an FCGR. Thus each FCGR represents the occurrence of oligonucleotides in the whole sequence. For dinucleotides (*k* = 2) the binary square is divided into a 4 × 4 grid, for trinucleotides (*k* = 3) into an 8 × 8 grid, and in general into a 2*^k^* × 2*^k^* grid. Figure 1C shows an FCGR (*k* = 3) of the whole *B. rapa* genome sequence.

If the nucleotide sequences differ in length, the resulting FCGRs will also differ in there overall frequencies. To overcome this sequence length bias each FCGR was standardized (Wang et al., 2005). If the FCGR is represented as for example a 2*^k^* × 2*^k^* matrix, the matrix $A={\left(a\right)}_{2^{k}\times 2^{k}}$ is transformed to a standardized FCGR as follows:

(3) $$Ā=\frac{4^{k}}{\overset{k}{\underset{i=1}{\sum }}\overset{k}{\underset{j=1}{\sum }}a_{i,j}}A$$

The nucleotide sequences of each data file (whole genome, EST, or mitochondrial genome data) were concatenated and the reverse complement of the concatenated sequence was appended. Characters other than “C,” “G,” “A,” or “T” were ignored. Some example FCGRs generated with *k* = 8 are shown in Figures 1D–L. Already by visual inspection it is obvious, that whole genome, EST, and mitochondrial genome FCGRs have distinct patterns (Figures 1D–F), while the FCGRs generated from the same data type of closely related species are very similar (Figures 1G–L). EST data disproportionately contain poly-A sequences, resulting in unusually high frequency values in the FCGRs. These subsequently dominate the distance matrix calculation for higher order FCGRs (*k* > 5) and misdirect the calculation of the phylogenetic trees (data not shown). Therefore, in the case of EST data, the two entries in each FCGR that contain poly-A and poly-T stretches were set to zero.

### Distances

In order to reveal the phylogenetic relation between the analyzed species we calculated pair-wise distances between the FCGRs. In general all distances that are applicable to matrices could be used. The following distances have already been described for comparing FCGRs: The Hamming distance (Campbell et al., 1999; Wang et al., 2005), the Euclidean distance (Edwards et al., 2002; Vinga and Almeida, 2003; Wang et al., 2005; Pandit and Sinha, 2010), the Image distance defined in Wang et al. (2005), and the Pearson distance (Almeida et al., 2001; Vinga and Almeida, 2003; Wang et al., 2005). Here, we chose the Pearson distance as a statistical distance and the Euclidean distance as a geometrical distance, which performed best in a comparison of difference distance methods (Wang et al., 2005). The Euclidean distance between two points in two-dimensional space is defined as the length of the line segment between these two points and can be calculated using the Pythagorean equation. This concept can be adapted to calculate the distance between two FCGRs. The Euclidean distance between two standardized FCGRs $A={\left(a\right)}_{2^{k}\times 2^{k}}$ and $B={\left(b\right)}_{2^{k}\times 2^{k}}$ is defined as follows:

(4) $$d_{\text{Euclidean}}\left(Ā,\bar{B}\right)=\frac{\sqrt{2^{k}}}{4^{k}}\sqrt{\overset{2^{k}}{\underset{i=1}{\sum }}\overset{2^{k}}{\underset{j=1}{\sum }}{\left(a_{i,j}-b_{i,j}\right)}^{2}}$$

The Pearson distance is based on a weighted Pearson correlation coefficient (Almeida et al., 2001; Wang et al., 2005). To calculate the Pearson distance, the FCGRs are represented as lists of the frequencies with *n* = 4*^k^* values. The Pearson distance between the non-standardized FCGRs *A* = (*x*_1_, …, *x_n_*) and *B* = (*y*_1_, …, *y_n_*) is defined as follows:

$$\begin{array}{l} nw=\overset{n}{\underset{i=1}{\sum }}x_{i}\cdot y_{i}\\ \bar{x}w=\frac{\overset{n}{\underset{i=1}{\sum }}x_{i}^{2}\cdot y_{i}}{nw},\;ȳw=\frac{\overset{n}{\underset{i=1}{\sum }}y_{i}^{2}\cdot x_{i}}{nw},\\ sx=\frac{\overset{n}{\underset{i=1}{\sum }}{\left(x_{i}-\bar{x}w\right)}^{2}\cdot x_{i}\cdot y_{i}}{nw},\;sy=\frac{\overset{n}{\underset{i=1}{\sum }}{\left(y_{i}-ȳw\right)}^{2}\cdot x_{i}\cdot y_{i}}{nw}\\ d_{\text{Pearson}}=1-\frac{\overset{n}{\underset{i=1}{\sum }}\frac{x_{i}-\bar{x}w}{\sqrt{sx}}\cdot \frac{y_{i}-ȳw}{\sqrt{sy}}\cdot x_{i}\cdot y_{i}}{nw}\text{(5)} \end{array}$$

### Generating phylogenetic trees

To generate the phylogenetic trees, pair-wise distance matrices were calculated for each *k* in 1, …, 0.8 with the Euclidean distance method as defined in Eq. 4 and the Pearson distance as defined in Eq. 5. The distance matrices were subjected to the Neighbor joining (NJ) and Fitch–Margoliash algorithms as implemented in the Phylip package^4^. Statistical support for branchings was obtained by applying the bootstrap re-sampling method. For each FCGR, 500 datasets were generated by random sampling with replacement. Based on these re-sampled FCGRs 500 phylogenetic trees were reconstructed for each *k* in 1, …, 0.8. The trees of each dataset were summarized to consensus trees using the *consense* program of the Phylip package. The topologies of the consensus trees were fixed and the branch lengths calculated with the Fitch–Margoliash algorithm. In the case of the NJ trees, a bootstrapped tree was chosen that had the same topology as the consensus tree and the bootstrap values were plotted onto this tree. The bootstrap values represent the percentage each interior branch has the same partition as the consensus tree.

### Generation of the reference tree for the whole genome analysis

For the reference tree of those species for which whole genome assemblies are available we identified, assembled, and annotated the sequences of the heterodimeric actin capping protein (CAP), α- and β-CAP, and the sequences of the actin-related proteins Arp2 and Arp3. The *B. rapa* and *Gossypium raimondii* genomes contain duplicates of these genes due to species-specific duplications. Therefore, only one of the duplicates had been used for the phylogenetic tree reconstructions. The CAP and Arp sequences were aligned, concatenated, and phylogenetic trees reconstructed using the NJ and the Maximum likelihood (ML) method. The NJ tree was unrooted and generated using ClustalW (Chenna et al., 2003) with standard settings and the Bootstrap (1,000 replicates) method. The ML tree was calculated using the JTT (Jones et al., 1992) substitution model as suggested by ProtTest (Darriba et al., 2011) with estimated proportion of invariable sites and bootstrapping (1,000 replicates) using RAxML (Stamatakis et al., 2008).

## Results

Phylogenetic trees based on whole genome, mitochondrial genome, and EST data were generated using the Euclidean or Pearson distance methods in combination with the NJ or the Fitch–Margoliash tree reconstruction algorithms. In order to reveal the influence of the lengths of the oligonucleotides we report trees of FCGRs generated with *k* = 3 (trinucleotides, 64 data points) and *k* = 8 (octanucleotides, 65,536 data points).

### Influence of sequence lengths on the phylogenetic trees

**Figure 2:**
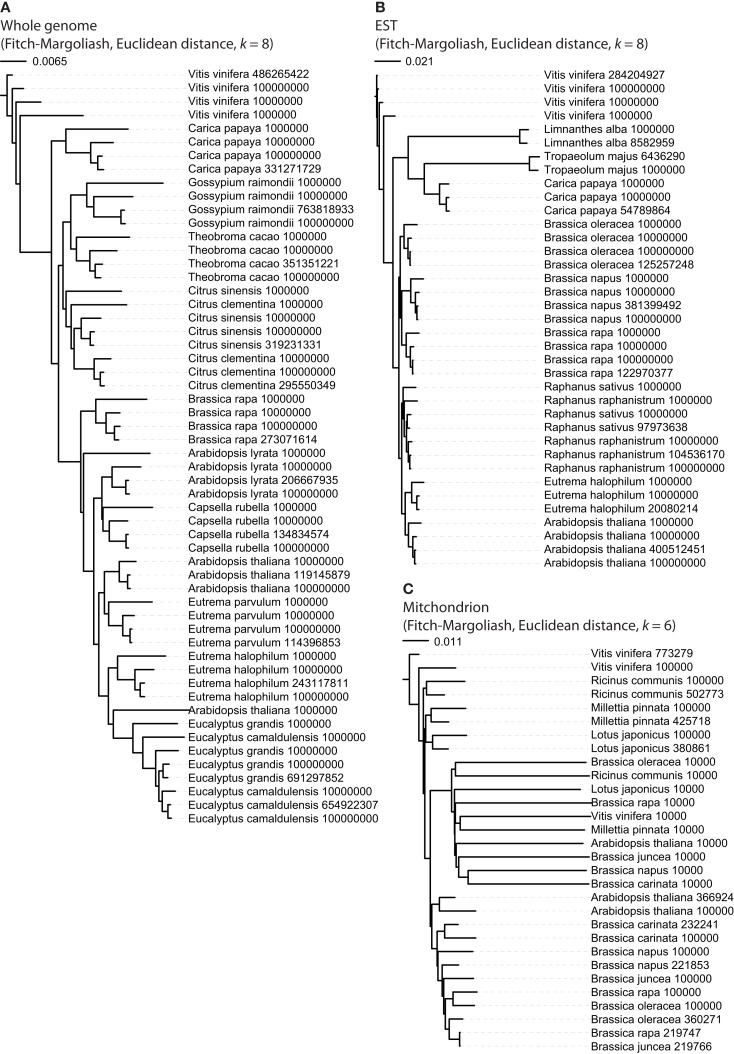
**Phylogenetic trees to reveal the potential influence of sequence length**. For each dataset sub-sequences with defined lengths were generated and FCGRs calculated. The lengths of the sequences were supposed to be sufficient for reliable tree reconstructions if the datasets generated from the same species grouped together. For whole genome **(A)** and EST **(B)** data 10,000,000 nt should be sufficient while the full-length mitochondrial genomes **(C)** are needed for reliable tree reconstructions.

First we tested whether different sequence lengths have an influence on the results (Figure 2). For the whole genome assemblies and the EST datasets, sub-sections of the sequences were generated with lengths of 10^6^, 10^7^, and 10^8^ nt. For that purpose the contigs or EST entries of each organism were shuffled, concatenated, and subsequently the sub-sequences generated by cutting the sequences at the respective positions. In the case of the whole genome data (Figure 2A), the FCGRs of the whole genome assemblies and the sub-sequences of each organism grouped together forming clusters. The only exceptions were the shortest 10^6^-nt sequences of *Citrus sinensis*, *Citrus clementina*, *Arabidopsis lyrata*, and *A. thaliana*, which group to different species. The FCGRs of the EST data group together for each species independently of the lengths of the sequences (Figure 2B). For the mitochondrial genomes datasets with shorter sequences of 10^4^ and 10^5^ nt were generated. Here the FCGRs of the 10^4^ nt sequences do not cluster together with those of the longer sequences of the corresponding species. The FCGRs of the mitochondrial sequences have been calculated based on hexanucleotides (*k* = 64,096 data points). Here, *k* = 6 was chosen, because in the case of higher *k* values (*k* = 7 or *k* = 8), the sequence length of the shortest sequences (10^4^ nt) would be less than the number of data points in the FCGRs. In the shortest sequences (10^4^ nt) many of the hexanucleotides are not covered at all resulting in many zero values for frequency positions, which lead to the unusual grouping of these FCGRs.

### Whole genome analysis

**Figure 3:**
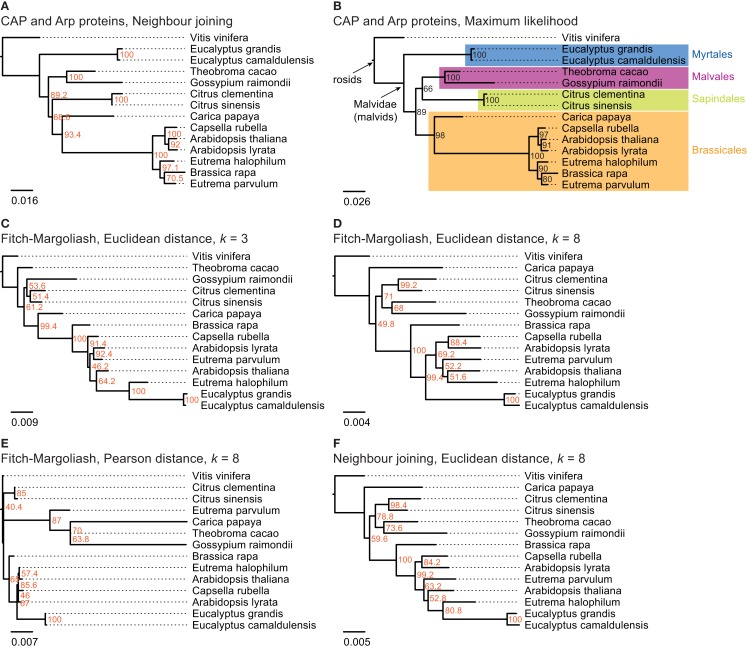
**The trees in (A,B) are based on a multiple sequence alignment of manually assembled CAP and Arp2/3 protein sequences**. The trees were calculated using the Neighbor joining and the Maximum likelihood method, respectively, with 1,000 bootstraps for each tree. In **(C–F)** phylogenetic trees were generated applying different methods on FCGRs of whole genome sequence data of species of the taxon Malvidae. In **(C–E)** the Fitch–Margoliash algorithm was used to calculate trees for 500 re-sampled datasets. Subsequently, a consensus tree was built and branch lengths were calculated based on the fixed consensus tree. The method used for the distance calculation and the resolution of the FCGRs are given on top of the trees. In **(F)** the Neighbor joining algorithm was used to calculate the tree.

In order to analyze the phylogenetic grouping of *B. rapa* in a whole genome context we searched for closely related plant species, for which whole genome assemblies are available. According to diArk (Hammesfahr et al., 2011), that comprises the most reliable and complete compilation of eukaryotic genome projects for which genome assemblies are available, the genomes of 13 different species (excluding different *A. thaliana* strains) of the taxon Malvidae have been sequenced and assembled: *A. lyrata* (Hu et al., 2011), *A. thaliana* (thale cress; Arabidopsis Genome Initiative, 2000), *B. rapa* subsp*. pekinensis* (Chinese cabbage; Wang et al., 2011), *Capsella rubella*, *Carica papaya* (Ming et al., 2008), *C. clementina*, *C. sinensis* (sweet orange), *Eucalyptus camaldulensis* (Murray red gum), *Eucalyptus grandis* (Flooded gum), *Eutrema halophilum* (salt cress), *Eutrema parvulum* (Dassanayake et al., 2011), *G. raimondii*, and *Theobroma cacao* (cacao plant; Argout et al., 2011). In addition, the genome of *Vitis vinifera* (grape vine; Jaillon et al., 2007; Velasco et al., 2007) was chosen as outgroup to root the trees. A species tree including all these organisms is not available. For comparison we therefore reconstructed trees of these species based on the alignment of the concatenated protein sequences of the actin CAP, Arp2, and Arp3 proteins (Figures 3A,B). The trees based on the NJ and ML methods are almost identical and differ only in the grouping of the two *Citrus* species (Sapindales clade) as independent clade (NJ, Figure 3A) or as sister clade of the Malvales (ML, Figure 3B). While the bootstrap support for all branchings is high, the support for the grouping of the *Citrus* clade is low in both trees (68.6% in the NJ and 66% in the ML tree, respectively). Both trees are in general agreement with phylogenetic analyses of the mitochondrial matR proteins (Zhu et al., 2007) and 61 chloroplast protein-coding genes (Bausher et al., 2006), and the combined analysis of 10 plastid and 2 nuclear (18S and 26S rDNA) genes (Cantino et al., 2007) that also show different groupings of the Sapindales clade. All trees agree with the grouping of the Malvales, Sapindales, and Brassicales into one clade and the grouping of the Myrtales as a sister clade, *C. papaya* being the most divergent of the analyzed Brassicales species and *C. rubella* being the closest relative of the *Arabidopsis* species. Except for the grouping of the two *Citrus* species the topology of the tree based on the ubiquitous cytoskeletal proteins CAP and Arp2/3 can thus be regarded as reference.

The resulting phylogenetic trees of the FCGRs differ as a function of data and methods used (Figures 3C–F). We reconstructed two trees based on the Euclidean distance and the Fitch–Margoliash algorithm but based on FCGRs with different resolution (*k* = 3 and *k* = 8 in Figures 3C,D, respectively), a tree using a different method for the distance calculation, the Pearson distance (Figure 3E), and a tree by applying a different method for the tree reconstruction, the NJ method (Figure 3F). In general, the trees agree with the reference tree except for the *Eucalyptus* species, which are either placed as sister group to *E. halophilum* (Figures 3C,F) or at the base of the Brassicales (Figures 3D,E) and thus far from their position according to the reference tree. In addition, *T. cacao* in Figure 2C, *C. papaya* in Figures 3D–F, and *E. parvulum* in Figure 2E are in wrong positions. None of the combinations of methods and data resulted in a correct resolution of the very closely related *Arabidopsis*, *Eutrema*, and *Capsella* species.

The tree based on the Pearson distance method (Figure 3E) contains the most deviations from the reference tree and this method therefore seems to be the least appropriate for reconstructing phylogenetic trees of whole genome sequences. This observation is in accordance with Wang et al. (2005). In addition, the bootstrap values do not provide reasonable support for most of the branchings except for the monophyly of the *Citrus* and the *Eucalyptus* clades. The trees based on high-resolution FCGRs (*k* = 8) using the Euclidean distance method (Figures 3D,F) have identical topologies except for the *Eucalyptus* outliers. In both trees *C. papaya* is placed as closest species to *V. vinifera* and not at the base of the Brassicales, *A. thaliana* grouped to the *Eutrema* species instead to its closest relative *A. lyrata*, and *B. rapa* is found at the base of the Brassicales instead of grouping to the *Eutrema* species. However, the misplacement of *Carica* and *A. thaliana* is not well supported (bootstrap values of 50–60%). Thus, the considerably faster NJ algorithm is a good alternative to the Fitch–Margoliash algorithm if run time is important. In contrast, the phylogenetic tree based on the low-resolution FCGRs (*k* = 3) contains more differences compared to the reference tree (Figure 3C).

### EST data analysis

**Figure 4:**
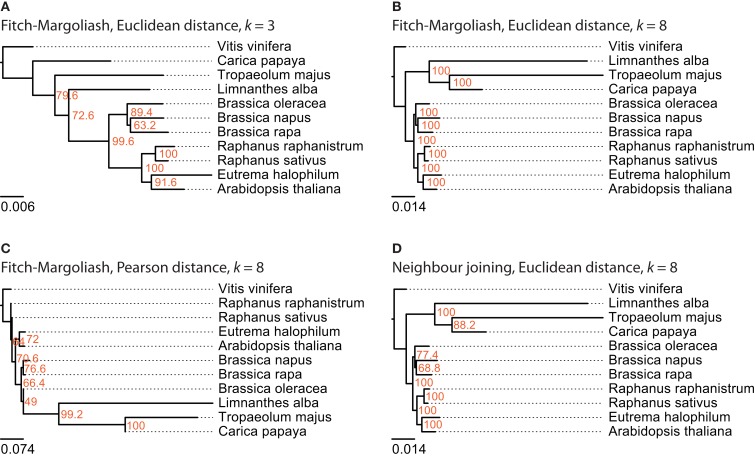
**The phylogenetic trees were generated applying different methods on FCGRs of public available EST data of the Brassicales taxon**. In **(A–C)** the Fitch–Margoliash algorithm was used to calculate trees for 500 re-sampled datasets. Subsequently, a consensus tree was built and branch lengths were calculated based on the fixed consensus tree. The methods used for the distance calculation and the resolution of the FCGRs are given on top of the trees. In **(D)** the Neighbor joining algorithm was used to calculate the tree.

For this analysis related species of *B. rapa* were chosen, for which more than 1,000 EST entries are available in the EST database of NCBI. There are ten species that belong to the Brassicales taxon and match this criteria: *A. thaliana*, *Brassica napus*, *Brassica oleracea*, *B. rapa*, *C. papaya*, *E. halophilum*, *Limnanthes alba*, *Raphanus raphanistrum*, *Raphanus sativus*, and *Tropaeolum majus* (Table 1). Again, *V. vinifera* was included as outgroup. The trees reconstructed from the FCGRs of the EST datasets are shown in Figure 4. The tree based on the Pearson distance and calculated with the Fitch–Margoliash algorithm (Figure 4C) shows many deviations from the known relationships of the species but also low support for the branchings. Like for the whole genome analysis, the Pearson distance concept is not appropriate for the reconstruction of reliable phylogenetic trees based on FCGRs. The trees based on the Euclidean distance (Figures 4A–D) have almost identical (low-resolution *k* = 3 compared to high-resolution data *k* = 8) to identical topologies (Fitch–Margoliash compared to NJ algorithm). Especially the species of the Brassicaceae clade are well resolved and their topology is highly supported in all trees. The Limnanthaceae, Tropaeolaceae, and Caricaceae are sister groups of the Brassicaceae. To our knowledge there is no highly resolved tree of these groups available that we could use as reference. Based on our experience with the whole genome data we suppose that the trees based on high-resolution data represent the more reliable topologies.

### Mitochondrial genome analysis

**Figure 5:**
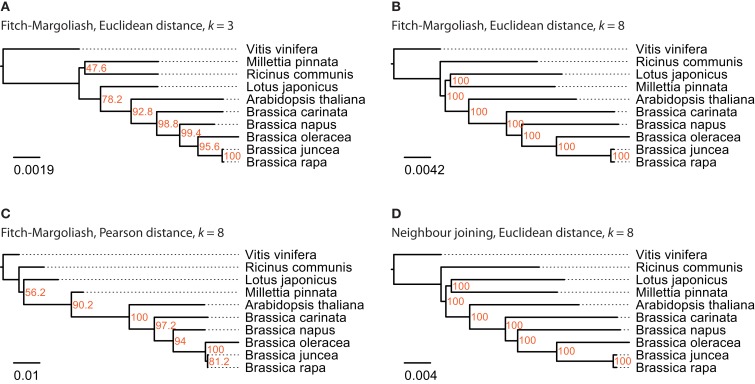
**The phylogenetic trees were generated applying different methods on FCGRs of available mitochondrial genome sequence data of the Rosids taxon**. In **(A–C)** the Fitch–Margoliash algorithm was used to calculate trees for 500 re-sampled datasets. Subsequently, a consensus tree was built and branch lengths were calculated based on the fixed consensus tree. The method used for the distance calculation and the resolution of the FCGRs are given on top of the trees. In **(D)** the Neighbor joining algorithm was used to calculate the tree.

For this analysis close relatives of *B. rapa* were chosen, for which sequenced mitochondria are available from NCBI. There were nine species in the Rosids taxon, whose mitochondrial genome sequences were available: *A. thaliana*, *Brassica carinata*, *Brassica juncea*, *B. napus*, *B. oleracea*, *B. rapa*, *Lotus japonicus*, *Millettia pinnata*, and *Ricinus communis* (Table 1). The mitochondrial genome of *V. vinifera* was used as outgroup. In contrast to the analyses of the other datasets, the trees based on the FCGRs of the mitochondrial genomes were very similar for the four different methods (Figure 5). Especially the sub-branches containing the five closely related *Brassica* species show exactly the same topology supported by high bootstrap values. While the topology of the Brassicales subfamily tree is well resolved the grouping of the Fabales *L. japonicus* and *M. pinnata* and the Malpighiales *R. communis*, which all belong to the fabids, is different in the four trees. Here, the trees based on the Euclidean distance with high-resolution FCGRs (*k* = 8) have the same well supported topology grouping the Fabales together (Figures 5B,D) independently which method has been used for the tree reconstruction. This is in agreement with the results from the whole genome and EST analysis that the use of FCGRs with high-resolution results in more reasonable trees, and that the Euclidean method for the calculations of the distances is more appropriate than the Pearson method.

### Computational resource comparison

The algorithm to calculate the CGRs and FCGRs has linear time complexity *O*(*L*) and space constant complexity *O*(1), where *L* is the length of the nucleotide sequence. In the case of whole genomes, the calculation of the CGRs and FCGRs took about 7,600 s for each genome, for EST data 2,800 s for each species, and 140 s for each mitochondrial genome. The time the algorithm needs to calculate the phylogenetic trees mainly depends on the distance matrix calculated for each species against each other species. This calculation has time complexity *O*(4*^k^s*^2^) and space complexity *O*(*s*^2^), where *s* is the number of species and *k* is the length of the oligonucleotide. The reconstructions of the phylogenetic trees took 98 s for *k* = 8 and the whole genome datasets (*k* = 7: 41 s, *k* = 6: 10 s, *k* = 3: 4 s), 86 s with *k* = 8 for the EST datasets (*k* = 7: 22 s, *k* = 3: 2 s) and 58 s for *k* = 8 and the mitochondrial genome datasets (*k* = 7: 13 s, *k* = 3: 1 s). These values refer to one round of bootstrapping. For comparison, one of the fastest whole genome alignment tools, called Mugsy, needs 45,000 s (ca. 12 h) to align the human and the mouse genomes (Angiuoli and Salzberg, 2011). However, whole genomes can only be aligned if they are from closely related species and, to our knowledge, phylogenies of multiple sequence alignments of the whole genomes from different eukaryotes have not been reconstructed yet.

## Discussion

In general, phylogenetic trees of species are reconstructed from amino acid or nucleotide sequence data, by comparing morphological characteristics, or by combining these data. While most of the sequence-based analyses are built on single genes, concatenated sequences are increasingly used, which could consist of even whole transcriptomes (phylogenomics). Here, we wanted to reconstruct the phylogeny of selected Brassicales species based on alignment-free sequence data. As approach we chose CGRs, which are scale-independent representations for genomic sequences (Jeffrey, 1990). Because CGRs are unique fingerprints of the corresponding sequences they cannot be compared directly. To reconstruct phylogenetic trees we therefore generated FCGRs at different resolutions. For the calculation of the distances between FCGRs we used the Euclidean (a geometric distance) and the Pearson (a statistical distance) method, and trees were reconstructed with the Fitch–Margoliash and the NJ algorithm.

Because of their different characteristics we compared three types of nucleotide sequences, nuclear genome sequences, mitochondrial genome sequences, and EST reads. Nuclear and mitochondrial genomes have been shown to have different GC contents and codon usage patterns (Zhang et al., 2007). EST data just comprise the exons and thus only part of the nuclear genome sequences. In addition EST data are potentially biased toward highly abundant genes and 5′- and 3′-terminal sequences. In order to reduce this bias we decided to include only those species for which at least 1,000 EST clones were available. Unfortunately, appropriate species from the Brassicales clade are not available for which all three types of nucleotide data have been sequenced. Therefore, we compared different sets of species for the three data types. Also, it is not known whether the mitochondrial genome data have been extracted from the whole genome datasets. As most of these are denoted as “draft assembly” we assume that the whole genome datasets still contain mitochondrial data. However, because of the very small size of the mitochondrial genomes compared to the nuclear genomes the results should be identical to those obtained from pure nuclear genome data. We would have liked to compare the results of each type of nucleotide sequence with the results of combined datasets but appropriate sequence data is not available. However, the EST and mitochondrial data do not comprise 1% of the whole genome data (Table 1) and a combined analysis should therefore be dominated by and be identical to the whole genome data.

The mitochondrial and whole genomes of the analyzed Brassicales species are of considerably different size, and different amounts of EST data are available. FCGRs naturally depend on the presence and frequency of the respective oligonucleotides and thus on the length of the analyzed sequence. For a reasonable result it is therefore essential to find the best balance between sequence length and FCGR resolution (oligonucleotide length), which represents the number of data available for the tree calculations and is also the main determinant for computing time. To exclude that the lengths of the concatenated sequences have an influence on the phylogenetic tree reconstructions of the Brassicales species at high FCGR resolution we calculated trees including the full-lengths sequences and specific defined subsets (Figure 2). At the resolution of octanucleotides, all partial sequences of whole genome assemblies containing more than 10 million nucleotides of each species group together while sets with 1 million nucleotides result in the ambiguous grouping of some species. In contrast, one million nucleotides of EST data, which correspond to the exon sequences, already result in consistent monophyly of all datasets of each species. Remarkably, this holds even true for the closely related *Brassica* species. The mitochondrial genomes of the analyzed species have sizes of 220–780 kbp. Thus, at the resolution of hexanucleotides it is not surprising that many oligonucleotides do not exist in sub-sections of 10 kbp leading to the artificial attraction of all these datasets in the reconstructed tree. Also, datasets of 100 kbp of the different *Brassica* species do not consistently group to the full-length mitochondrial genomes. Therefore, for mitochondrial data the resolution has to be reduced or full-length data to be used. As outgroup we choose *V. vinifera* in all analyses.

According to the diArk database, whole genome assemblies are available for 34 species belonging to the Malvidae/malvids (Hammesfahr et al., 2011). Twenty-two of them are *A. thaliana* strains of which we only included the reference strain into the analysis. A species tree including all these sequenced Malvidae is not available. Therefore, we assembled and annotated the CAPs α- and β-CAP, and the actin-related proteins Arp2 and Arp3 to generate a reference tree. The CAP and Arp proteins have been chosen for the reference tree because they are ubiquitous and well conserved in all eukaryotes (Goley and Welch, 2006; Cooper and Sept, 2008), and duplicates were most probably removed after the many whole genome duplication events that happened in plant evolution (Van de Peer, 2011). For example, the *A. thaliana* genome has experienced two duplications since its divergence from *Carica* (Tang et al., 2008), but has retained single copies of the CAP and Arp genes (Hammesfahr and Kollmar, 2012). Nevertheless, duplicated CAP and Arp2/3 genes have been identified in the *B. rapa* and *G. raimondii* genomes that are, however, the result of species-specific duplications. Only one of each duplicate has been used in this analysis. The phylogenetic tree of the concatenated CAP and Arp proteins is in agreement with other recent analyses containing part of the species (Bausher et al., 2006; Zhu et al., 2007; Wang et al., 2009) and can thus be regarded as reference tree. Compared to this reference tree, the FCGR tree based on the Pearson distance displays the most discrepancies followed by the tree based on low-resolution data (*k* = 3, trinucleotides). In addition, most of the branchings have low bootstrap values. The trees based on high-resolution data (*k* = 8,65,536 data points) and the Euclidean distance method show overall agreement with the reference trees independent of the method used for the tree reconstruction. Notably, *C. papaya* and *B. rapa* group wrongly, although both are only shifted by one branching event. Most surprisingly, the *Eucalyptus* species are completely wrongly grouped in all FCGR trees. Their exclusion from the tree calculation did not change the grouping of the other species (data not shown). However, the grouping of the Myrtales branch, which contains the *Eucalyptus* species, is different in all published trees (Bausher et al., 2006; Zhu et al., 2007; Wang et al., 2009) and their wrong placement in the FCGR trees might be due to some unknown characteristics of the genomes. Probably, they would group better, if species from other branches like the Crossosomatales, Geraniales, and Fabidae branches were included in the analysis. The phylogenetic trees of the FCGRs of the mitochondrial genomes are very similar independently of the resolution, distance measure, and tree reconstruction method. Therefore either the species selection was fortunate or mitochondrial genome data is less sensitive with respect to these parameters.

When working with the EST data we observed disproportionate high frequencies for poly-A and poly-T oligonucleotides in the FCGRs. Probably, the poly-A tails were not consistently removed during the cDNA library construction. For low-resolution data (up to *k* = 5) the differences of the frequencies of these oligonucleotides to the next-highest values were not large enough to considerably bias the phylogenetic tree reconstructions. However, the topologies of trees based on high-resolution data (*k* > 5) are strongly disturbed. Therefore, we set the values for the frequencies of the poly-A and poly-T oligonucleotides to zero before we started the tree calculations. The artificial oligonucleotides generated at the boundaries of the concatenated EST reads apparently do not influence the resulting trees. The phylogeny of the *Brassica* species is slightly different compared to that obtained from the mitochondrial genome data. The genus *Brassica* includes 41 species (Velasco and Fernández-Martínez, 2010) the six with the highest economic importance being *B. rapa* (A), *Brassica nigra* (B), *Brassia oleracea* (C), *B. napus* (AC), *B. juncea* (AB), and *B. carinata* (BC). The first three comprise the three elementary species while the other three are amphidiploids that originated from natural hybridizations between two of the elementary species (Velasco and Fernández-Martínez, 2010). Thus the amphidiploid EST data contain mixtures of the hybridized species and dependent on which part is overrepresented in the data they will look closer related to one of their parent species. Although the distance in the phylogenetic tree is very small, *B. napus* seems to be closer to *B. rapa* based on the mitochondrial data. Based on the EST data, the hybrids *B. juncea* and *B. carinata* are more divergent than the parent species *B. rapa* and *B. oleracea*. Probably the part of the more divergent parent species *B. nigra* is dominating in this case.

In general we could show that FCGRs are well suited to phylogenetically group plant genomes and exonomes from even closely related species. We assume that FCGRs could also be used to group all eukaryotes provided that a balanced set of species from all lineages is taken. This has in part already been demonstrated on the phylogeny of 26 mitochondrial genomes of which only three were placed completely wrong when using the Euclidean distance method (Wang et al., 2005). However, this analysis was solely based on data from mitochondrions and biased against fish and mammalian species. Our analysis of the Brassicales clade has shown that high-resolution data (octanucleotides and longer sequences) result in better tree topologies and higher support for branchings. Trees based on the Pearson distance, which is a statistical distance measure, are less reliable than those based on Euclidean distances. The Fitch–Margoliash and NJ algorithms result in similar to identical trees. We have shown for the first time that the bootstrap concept to determine the support of the branchings in the tree, which is well established for trees based on sequence alignments since decades (“taxon-by-character” data matrix; Felsenstein, 1985), can also be applied to trees based on FCGRs. In another study it has been shown that although longer word lengths could reveal the correct clustering of the HIV-I subtypes in contrast to shorter word lengths (Pandit and Sinha, 2010) the grouping within the subtypes was always different. Also in this case a bootstrap analysis could have helped in the interpretation of the various branchings and we would recommend applying the bootstrap concept to all phylogenies based on FCGRs. FCGRs are fast to calculate and could be used in combination with alignment based data and morphological characteristics to improve the phylogenetic classification in ambiguous cases.

## Conflict of Interest Statement

The authors declare that the research was conducted in the absence of any commercial or financial relationships that could be construed as a potential conflict of interest.

## Footnotes

^1^http://www.diark.org

^2^http://www.w3.org/Graphics/SVG

^3^http://ruby-lang.org

^4^http://evolution.genetics.washington.edu/phylip.html
